# Edentulousness and the Likelihood of Becoming a Centenarian: Longitudinal Observational Study

**DOI:** 10.2196/68444

**Published:** 2025-03-21

**Authors:** Xindi Wei, Longfei Zhuang, Yuan Li, Junyu Shi, Yijie Yang, Hongchang Lai, Beilei Liu

**Affiliations:** 1 Shanghai Ninth People's Hospital, School of Medicine, Shanghai Jiao Tong University, 639 Zhizaoju Road, Shanghai, 200011, China, 86 02123271699; 2 Huishang Dental Clinic, Shanghai, China

**Keywords:** public health, edentulous, oral-systemic disease, epidemiology, cohort studies

## Abstract

**Background:**

In recent decades, the global life expectancy has risen notably to approximately 73.5 years worldwide, coinciding with a rapid growth in the older adult population, which presents a significant public health challenge in promoting healthy aging and longevity.

**Objective:**

This study aimed to prospectively investigate the link between edentulousness and the likelihood of reaching centenarian status among individuals aged 80 years and older.

**Methods:**

Data from the Chinese Longitudinal Healthy Longevity Survey were analyzed. Logistic regression models were used to assess the relationship between edentulousness and the likelihood of becoming a centenarian. Demographic characteristics, lifestyle habits, and disease histories were adjusted as confounding factors. Several sensitivity analyses, including propensity score matching and 2-year lag analyses, were conducted to further assess the association between edentulousness and the likelihood of becoming a centenarian. The correlation between the number of natural teeth as a continuous variable and the likelihood of becoming a centenarian was evaluated as well.

**Results:**

The study included 4239 participants aged 80-100 years. After adjusting for all covariates, the likelihood for becoming a centenarian increased in the nonedentulous group compared to the edentulous group (odds ratio [OR] 1.384, 95% CI 1.093‐1.751). The relationship persisted after propensity score matching analysis (OR 1.272, 95% CI 1.037‐1.561). The association remained statistically significant after excluding participants with a follow-up duration of less than 2 years (OR 1.522, 95% CI 1.083‐2.140; *P*=.02). Furthermore, a significant positive association between the number of natural teeth and the likelihood of becoming a centenarian was found after adjusting for all covariates (OR 1.022, 95% CI 1.002‐1.042; *P*=.03), which aligned with the main results of the study.

**Conclusions:**

The findings revealed that the presence of natural teeth was linked to an increased probability of becoming a centenarian, underscoring the importance of maintaining oral health even in advanced age.

## Introduction

Over the past few decades, the global life expectancy at birth has significantly risen to approximately 73.5 years worldwide and 77.6 years in mainland China [1,2]. Additionally, the proportion of individuals aged 65 years and older is rapidly expanding, constituting 13.5% of the population in China [3]. This increase in life expectancy has coincided with a rapid expansion of the aging population, presenting a substantial public health challenge in fostering healthy aging and longevity. However, focusing solely on traditional determinants of longevity is insufficient, underscoring the importance of identifying other modifiable risk factors.

Oral diseases are widespread globally, affecting over 3.5 billion individuals [4,5], yet their influence on longevity is often overlooked. In addition to sociodemographic, genetic, and lifestyle factors [6], tooth loss—the ultimate outcome of oral diseases and a surrogate for overall health status—is essential for healthy aging as well. The retention of natural teeth over a lifetime is a fundamental aspect of overall well-being [7]. Tooth loss can be a potential risk factor for longevity that is independently related to the onset of disability and mortality in old age [7-9]. For instance, the 6-year mortality rate of individuals with edentulousness (the lack of teeth) who do not use dentures was significantly higher than that of the individuals with ≥20 teeth [10]. Another study based on Baltimore Longitudinal Study of Aging indicated that being edentulous or having fewer than 20 teeth was independently associated with the mortality of older adults [11]. Centenarians comprise those who successfully age and have good resilience, and the number of centenarians is increasing worldwide [12-14]. Centenarians and their offspring demonstrated better oral health, suggesting the potential relationship between natural teeth retention and longevity [8]. Moreover, possessing ≤20 natural teeth was an independent risk factor for frailty among centenarians [3]. However, there is no cohort study that focuses on the impact of natural teeth retention on achieving centenarian status; thus, leveraging aging-focused, big data analytics to understand the intricate relationship among the older adult population becomes imperative.

The objective of this study was to prospectively investigate the association between edentulousness and the likelihood of becoming a centenarian in individuals aged 80 years and older, using data from the Chinese Longitudinal Healthy Longevity Survey (CLHLS)—an aging-focused, nationally representative cohort of the older Chinese population. These findings have the potential to inform interventions aimed at maximizing life expectancy.

## Methods

### Study Design and Population

The CLHLS is a comprehensive nationwide study that uses random sampling techniques to select participants from half of the counties and cities in 22 out of the 31 provinces across mainland China, covering approximately 85% of the total population [15]. The CLHLS collected data from 8 waves of surveys carried out in 1998, 2000, 2002, 2005, 2008, 2011, 2014, and 2018. Each survey round involved follow-ups with existing participants and the recruitment of new participants.

This cohort study used the baseline data from 1998, and the mortality follow-up data were from 1998, 2000, 2002, 2005, 2008, 2011, 2014, and 2018. As long as the individuals were aged 80 years or older in 1998, they could potentially live to 100 years in 2018. Thus, 6675 participants (aged ≥80 years and <100 years) who had the potential to age to 100 years or older were first included in this study. The participants who were lost to follow-up or had incomplete information on confounders were then excluded, and 4239 participants were included in the final analyses.

This study adheres to the STROBE (Standards for Reporting of Observational Studies in Epidemiology) guidelines [16].

### Exposure Variable

The number of natural teeth of the participants was collected in the survey. Participants were divided into two categories based on the existence of natural teeth. The edentulous group was defined as participants with the complete loss of all dentition, while the nonedentulous group was defined as participants with at least 1 tooth.

### Outcome Variable

Interviews were conducted with a close family member of the participants who had passed away between the previous wave’s interview and the subsequent survey. The year of death and the age at death were collected. Individuals reaching the age of 100 years were defined as centenarians.

### Covariates

Age in 1998, sex (male or female), ethnicity (Han Chinese or other), residence (urban or rural), marital status (currently married and living with a spouse, widowed, separated, divorced, or never married), exercise habits (yes or no), smoking status (nonsmoker, former smoker, or current smoker), alcohol use (nondrinker, former drinker, or current drinker), denture use (yes or no), diabetes (yes or no), hypertension (yes or no), stroke or cardiovascular disease (CVD; yes or no), and cancer (yes or no) were selected as covariates [6]. Denture use included the use of complete dentures and removable partial dentures [17]. All data were obtained by a face-to-face interview via a questionnaire by well-trained interviewers from the local Centers for Disease Control and Prevention.

### Statistical Analysis

In the descriptive statistics, median and IQR were used for continuous variables, and frequency distributions were used for categorical variables. Continuous variables were compared by ANOVA tests for variables meeting the assumptions of normal distribution and homogeneity of variance, and by Kruskal-Wallis H test for those not meeting these assumptions. Categorical variables were compared using *χ*^2^ tests. The univariate and multivariate logistic regression models were adopted to evaluate the odd ratios (ORs) and 95% CIs pertaining to the association between edentulousness and the likelihood of becoming a centenarian. To control for confounders, three logistic regression models were constructed to eliminate the influence of covariates. No variables were adjusted in model 1. Model 2 adjusted for the age in 1998, sex, ethnicity, marital status, smoking status, alcohol use, and exercise habits. Model 3 further incorporated adjustments for denture use, diabetes, hypertension, stroke or CVD, and cancer. Several sensitivity analyses were performed. First, a 1:1 propensity score matching (PSM) analysis was conducted to balance the differences between the edentulous and nonedentulous groups using the *MatchIt* R package [18], which adjusted for the age in 1998, sex, ethnicity, marital status, exercise habits, smoking status, alcohol use, denture use, diabetes, hypertension, stroke or CVD, and cancer. The data after PSM were then analyzed using logistic regression to confirm the association between edentulousness and the likelihood of becoming a centenarian, with only the covariates that were still significant after PSM being adjusted. Second, a 2-year lag analysis was conducted by excluding individuals whose follow-ups were less than 2 years. Third, the association between the number of natural teeth as a continuous variable and the likelihood of becoming a centenarian was explored by logistic regression models.

A 2-sided *P* value <.05 was considered statistically significant in all analyses. All statistical analyses were performed by R (version 4.3.1; R Foundation for Statistical Computing).

### Ethical Considerations

Ethical approval was granted by the Biomedical Ethics Committee of Peking University (IRB00001052-13,074), and all participants or their proxy respondents provided informed consent without receiving financial compensation. The data have been anonymized to protect the privacy of participants.

## Results

### Baseline Characteristics of the Study Sample

**Figure 1.**
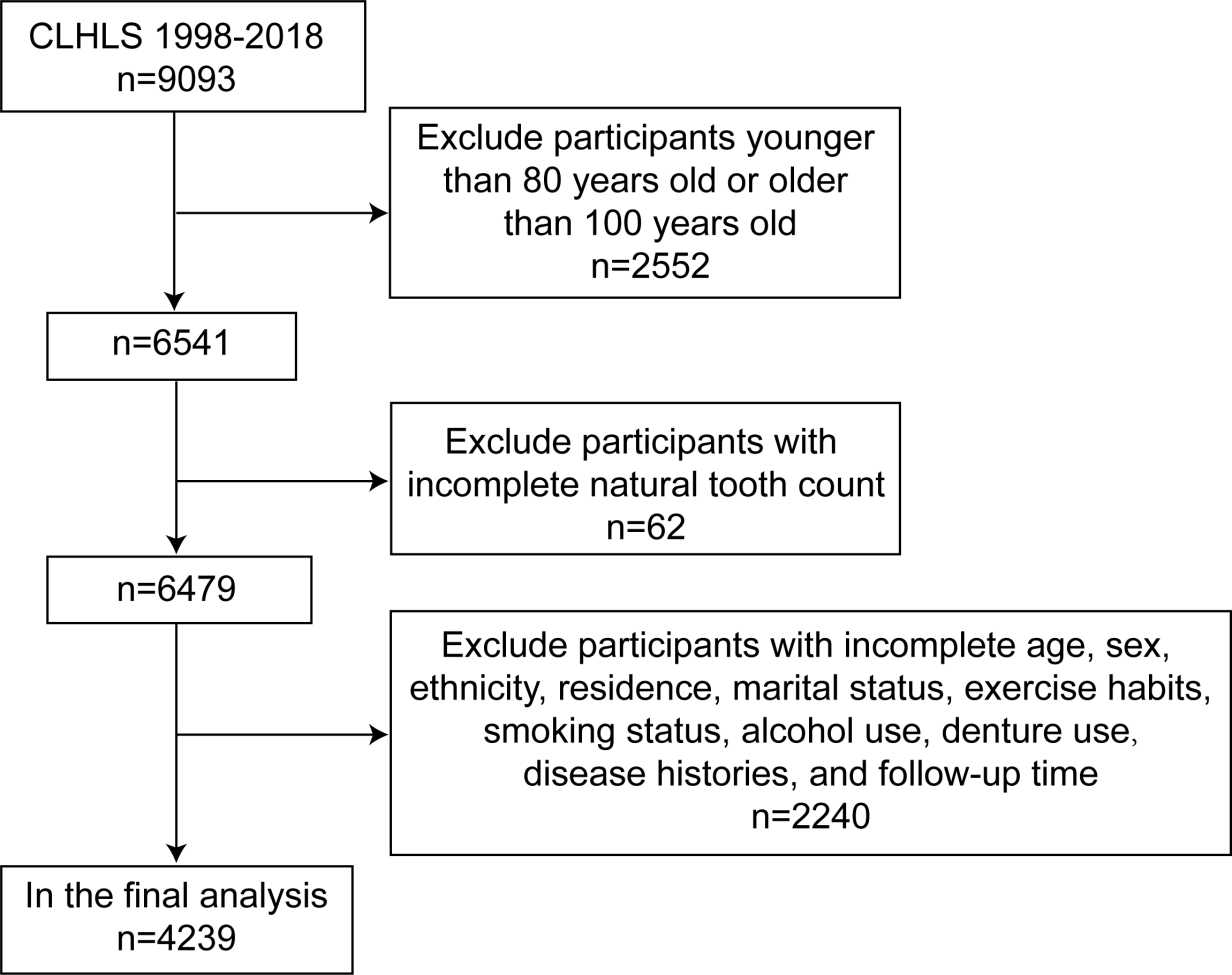
Flowchart of the process for the selection of eligible participants. CLHLS: Chinese Longitudinal Healthy Longevity Survey.

As depicted in Figure 1, this study included 4239 participants from the CLHLS 1998‐2018. Table 1 demonstrated the baseline characteristics of these participants. Significant differences were detected among the participants in various factors, including age (*P*<.001), sex (*P*<.001), ethnicity (*P*=.03), denture use (*P*<.001), smoking status (*P*<.001), alcohol use (*P*<.001), and hypertension (*P*=.02). There were no significant differences between the two groups in terms of residence (*P*=.09), exercise habits (*P*=.87), diabetes (*P*>.99), stroke or CVD (*P*=.17), and cancer (*P*=.47). Overall, 607 (14.3%) of the 4239 individuals became centenarians during follow-up, and 264 (43.5%) of these 607 centenarians were edentulous.

**Table 1.** Characteristics of the participants (n=4239).

Characteristics	Noncentenarians (n=3632)	Centenarians (n=607)	Statistic (*df*)	*P* value
Age (years), median (IQR)	88 (84-93)	98 (96-99)	1056.900 (1)	<.001^a^
Sex, n (%)			40.334 (1)	<.001^b^
Male	1795 (49.4)	215 (35.4)		
Female	1837 (50.6)	392 (64.6)		
Ethnicity, n (%)			4.596 (1)	.03^b^
Han Chinese	3336 (91.9)	541 (89.1)		
Other	296 (8.1)	66 (10.9)		
Residence, n (%)			2.796 (1)	.09^b^
Urban	540 (14.9)	74 (12.2)		
Rural	3092 (85.1)	533 (87.8)		
Marital status, n (%)			40.658 (4)	<.001^b^
Currently married and living with a spouse	759 (20.9)	62 (10.2)		
Separated	51 (1.4)	8 (1.3)		
Divorced	22 (0.6)	3 (0.5)		
Widowed	2751 (75.7)	529 (87.1)		
Never married	49 (1.3)	5 (0.8)		
Exercise habits, n (%)			0.027 (1)	.87^b^
Yes	990 (27.3)	168 (27.7)		
No	2642 (72.7)	439 (72.3)		
Smoking status, n (%)			32.136 (2)	<.001^b^
Current smoker	799 (22)	78 (12.9)		
Former smoker	600 (16.5)	89 (14.7)		
Nonsmoker	2233 (61.5)	440 (72.5)		
Alcohol use, n (%)			21.517 (2)	<.001^b^
Current drinker	943 (26)	120 (19.8)		
Former drinker	441 (12.1)	52 (8.6)		
Nondrinker	2248 (61.9)	435 (71.7)		
Self-reported diabetes, n (%)			<0.001 (1)	>.99^b^
Yes	23 (0.6)	4 (0.7)		
No	3609 (99.4)	603 (99.3)		
Self-reported hypertension, n (%)			5.042 (1)	.02^b^
Yes	502 (13.8)	63 (10.4)		
No	3130 (86.2)	544 (89.6)		
Self-reported stroke or CVD^c^, n (%)			1.849 (1)	.17^b^
Yes	133 (3.7)	15 (2.5)		
No	3499 (96.3)	592 (97.5)		
Self-reported cancer, n (%)			0.528 (1)	.47^b^
Yes	17 (0.5)	1 (0.2)		
No	3615 (99.5)	606 (99.8)		
Edentulousness, n (%)			23.847 (1)	<.001^b^
Yes	1206 (33.2)	264 (43.5)		
No	2426 (66.8)	343 (56.5)		
Denture use, n (%)			15.562 (1)	<.001^b^
Yes	784 (21.6)	88 (14.5)		
No	2848 (78.4)	519 (85.5)		

a Kruskal-Wallis H test.

b Chi-square test.

c CVD: cardiovascular disease.

### Correlation Between Edentulousness and the Likelihood of Becoming a Centenarian

A significant association between edentulousness and the likelihood of becoming a centenarian was found among the participants. After adjusting for all covariates in model 3, the likelihood for becoming a centenarian increased in the nonedentulous group compared to the edentulous group (odds ratio [OR] 1.384, 95% CI 1.093‐1.751; *P*=.007; Table 2).

**Table 2.** Association between edentulousness and the likelihood of becoming a centenarian.

Edentulousness	Participants (N=4239), n (%)	Model 1^a^		Model 2^b^		Model 3^c^	
		OR^d^ (95% CI)	*P* value	OR (95% CI)	*P* value	OR (95% CI)	*P* value
Yes	1470 (34.7)	Ref^e^		Ref		Ref	
No	2769 (65.3)	0.646 (0.542-0.769)	<.001	1.311 (1.042-1.648)	.02	1.384 (1.093-1.751)	.007

a Model 1: unadjusted.

b Model 2: model 1 plus additional adjustment for the age in 1998, sex, ethnicity, marital status, smoking status, alcohol use, and exercise habits.

c Model 3: model 2 plus additional adjustment for denture use and disease histories (including diabetes, hypertension, stroke or cardiovascular disease, and cancer).

d OR: odds ratio.

e Ref: reference.

### Sensitivity Analyses

The robustness of the observed association was confirmed in the sensitivity analyses. PSM analysis was used to further evaluate the association between edentulousness and the likelihood of becoming a centenarian. Participants were divided into two groups for PSM analysis—the edentulous group and the nonedentulous group—and nearly all covariates were not significantly different after PSM (Multimedia Appendix 1). A significant increase in the likelihood of becoming a centenarian in the nonedentulous group was observed compared to the reference group (OR 1.272, 95% CI 1.037‐1.561; *P*=.02; Table 3). Even after excluding participants who were followed up for less than 2 years, the association remained significant (OR 1.522, 95% CI 1.083‐2.140; *P*=.02; Table 4). Regarding the association between the number of natural teeth as a continuous variable and the likelihood of becoming a centenarian, a notable positive association was found after adjusting for all covariates (OR 1.022, 95% CI 1.002‐1.042; *P*=.03; Multimedia Appendix 2), which was comparable with the main results of this study.

**Table 3.** Association between edentulousness and the likelihood of becoming a centenarian after propensity score matching.

Edentulousness	Participants (n=2560), n (%)	OR^a^ (95% CI)	*P* value
Yes	1280 (50)	Ref^b^	
No	1280 (50)	1.272 (1.037-1.561)	.02

a OR: odds ratio.

b Ref: reference.

**Table 4.** Association between edentulousness and the likelihood of becoming a centenarian in a 2-year lag analysis.

Edentulousness	Participants (n=2310), n (%)	Model 1^a^		Model 2^b^		Model 3^c^	
		OR^d^ (95% CI)	*P* value	OR (95% CI)	*P* value	OR (95% CI)	*P* value
Yes	410 (17.7)	Ref^e^		Ref		Ref	
No	1900 (82.3)	0.712 (0.572-0.885)	.002	1.414 (1.017-1.966)	.04	1.522 (1.083-2.140)	.02

a Model 1: unadjusted.

b Model 2: model 1 plus additional adjustment for the age in 1998, sex, ethnicity, marital status, smoking status, alcohol use, and exercise habits.

c Model 3: model 2 plus additional adjustment for denture use and disease histories (including diabetes, hypertension, stroke or cardiovascular disease, and cancer).

d OR: odds ratio.

e Ref: reference.

## Discussion

### Principal Findings

In this large-scale cohort study, the association between edentulousness and the likelihood of becoming a centenarian among populations aged 80 years or older was comprehensively analyzed. The likelihood of becoming a centenarian increased for the nonedentulous group compared to the edentulous group. Importantly, the correlation persisted after PSM analysis and was confirmed by the 2-year lag analysis. A significant positive association was found between the number of natural teeth and the likelihood of becoming a centenarian, aligning with the primary findings of this investigation.

The outcomes of this study generally support previous research, suggesting that retaining natural teeth is linked to reduced mortality rates among individuals aged 80 years or older. A previous study indicated that a majority of centenarians expressed contentment with their oral health [19]. A cross-sectional study using data from the New England Centenarian Study unveiled a lower prevalence of tooth loss among centenarians compared to their peers at the ages of 65-74 years, hinting at tooth loss as a potential indicator of decreased longevity. Notably, both centenarians and their descendants exhibited superior oral health compared to the control group [8]. Another cross-sectional study that included 1034 centenarians from the CLHLS dataset indicated that having ≤20 natural teeth was an independent risk factor for frailty among centenarians [3], underscoring the significance of dental health for this age group. A population-based survey from Finland disclosed that even a small number of missing teeth could signify a heightened risk of overall mortality, emphasizing the link between tooth loss and mortality [20]. These findings align with the results of this study, and this research further enhances existing knowledge by showcasing that possessing natural teeth correlates with an increased likelihood of reaching centenarian status through extensive follow-ups and several sensitivity analyses. The robustness of this study was reaffirmed by a series of sensitivity analyses, as the association persisted after PSM analysis and was confirmed by the 2-year lag analysis. Upon examining the relationship between the number of natural teeth and the probability of attaining centenarian status, a significant positive correlation emerged. This association remained robust even after adjusting for all covariates, aligning with the principal findings of this study. These results underscore the importance of managing oral diseases, suggesting that older adults with natural teeth have a greater chance of becoming centenarians.

In this study, the OR changed from 0.646 to 1.384 from model 1 to model 3 for the correlation between edentulousness and the likelihood of becoming a centenarian, suggesting the existence of potential confounding factors. By adjusting the variables that were included in the model, age in 1998 was found to be the variable causing the OR to invert. Given that the outcome of this study was centenarian status, the age of the participants at enrollment could profoundly influence the outcome. Older individuals at enrollment were more likely to reach the age of 100 years. Therefore, to further mitigate this influence, a 2-year lag analysis was conducted in the *Sensitivity Analyses* section, excluding older individuals whose follow-ups were less than 2 years.

The precise mechanism underlying the higher likelihood of reaching centenarian status among individuals with natural teeth remains incompletely understood. Previous studies indicated an association between the number of teeth and masticatory function [21], and reduced chewing ability was related to premature death [22]. Individuals with edentulousness may have an unbalanced food selection, consuming inadequate amounts of fruits and vegetables, and their nutritional status may dispose these individuals to more chronic diseases [7,23], as the dietary intake pattern influences the microbial compositions and systemic inflammation [24].

This study demonstrates notable strengths by using longitudinal data from a sizable, nationally representative cohort of older Chinese individuals, enabling a prospective assessment of the link between edentulousness and achieving centenarian status in older adults. Furthermore, the study used various models that adjusted for multiple variables and conducted several sensitivity analyses to enhance the reliability of the results. Significantly, this research unveils, for the first time, the influence of edentulousness on the likelihood of becoming a centenarian. Nevertheless, this study is subject to various limitations. First, most lifestyle behaviors were self-reported, introducing potential measurement errors. Second, medical conditions were also self-reported, despite detailed explanations provided by trained interviewers during data collection. This method likely led to an underestimation of disease prevalence, possible misclassification, and residual confounding due to unmeasured medical conditions [6]. Third, despite adjusting for numerous confounding factors, the association could still be influenced by unmeasured or residual confounders, such as nutrition status that may affect both dental conditions and lifespan. Fourth, there may be reverse causality in this study. Individuals in better health status are more likely to retain their teeth, potentially biasing the interpretation of the findings. Finally, the survivorship bias may exist, as the participants included were aged ≥80 years at baseline, which may represent a population in relatively better health and may not be fully representative of all older adults.

### Conclusions

In this cohort study involving individuals aged 80 years or older in China, the presence of natural teeth was linked to an increased probability of reaching the age of 100 years, emphasizing the significance of preserving oral health even in advanced age. Implementing targeted intervention strategies for oral health to enhance overall well-being could potentially contribute to longevity. Further prospective research and basic research experiments are essential to validate these findings and illuminate the underlying mechanisms.

## Supplementary material

10.2196/68444 Multimedia Appendix 1 Characteristics of the participants after propensity score matching (n=2560).

10.2196/68444 Multimedia Appendix 2 Association between the number of natural teeth and the likelihood of becoming a centenarian.

## Abbreviations

CLHLS: Chinese Longitudinal Healthy Longevity Survey
CVD: cardiovascular disease
OR: odd ratio
PSM: propensity score matching
STROBE: Strengthening the Reporting of Observational Studies in Epidemiology
