# Supplementary material for: Edentulousness and the Likelihood of Becoming a Centenarian: Longitudinal Observational Study
Source: JMIR Aging. 2025 Mar 21;8:e68444. doi: 10.2196/68444 (PMC11951808; doi:10.2196/68444)
Supplement: Multimedia Appendix 1 [file aging-v8-e68444-s001.docx]

Multimedia Appendix1. Characteristics of the participants after PSM (n=2,560).

| Characteristics | Edentulousness | Non-edentulousness | Statistic | *P* value |
| --- | --- | --- | --- | --- |
| Age ^a^ | 98 (96, 99) | 90 (85, 94) | 0.598 | .383 ^c^ |
| Sex ^b^ |  |  | 8.727 | .003 ^d^ |
| Male | 513 (40.1) | 588 (45.9) |  |  |
| Female | 767 (59.9) | 692 (54.1) |  |  |
| Ethnicity ^b^ |  |  | 0.428 | .513 ^d^ |
| Han Chinese | 1182 (92.3) | 1172 (91.6) |  |  |
| Other | 98 (7.7) | 108 (8.4) |  |  |
| Residence ^b^ |  |  | 2.450 | .118 ^d^ |
| Urban | 173 (13.5) | 202 (15.8) |  |  |
| Rural | 1107 (86.5) | 1078 (84.2) |  |  |
| Marital status ^b^ |  |  | 8.256 | .083 ^d^ |
| Currently married and living with spouse | 193 (15.1) | 236 (18.4) |  |  |
| Separated | 14 (1.1) | 20 (1.6) |  |  |
| Divorced | 11 (0.9) | 9 (0.7) |  |  |
| Widowed | 1046 (81.7) | 992 (77.5) |  |  |
| Never married | 16 (1.2) | 23 (1.8) |  |  |
| Exercise ^b^ |  |  | 0.999 | .318 ^d^ |
| Yes | 336 (26.2) | 313 (24.5) |  |  |
| No | 944 (73.8) | 967 (75.5) |  |  |
| Smoking ^b^ |  |  | 4.078 | .130 ^d^ |
| Current smoker | 237 (18.5) | 273 (21.3) |  |  |
| Former smoker | 201 (15.7) | 211 (16.5) |  |  |
| Nonsmoker | 842 (65.8) | 796 (62.2) |  |  |
| Drinking ^b^ |  |  | 0.303 | .859 ^d^ |
| Current drinker | 277 (21.6) | 285 (22.3) |  |  |
| Former drinker | 134 (10.5) | 139 (10.9) |  |  |
| Nondrinker | 869 (67.9) | 856 (66.9) |  |  |
| Self-reported diabetes ^b^ |  |  | 0.212 | .645 ^d^ |
| Yes | 8 (0.6) | 11 (0.9) |  |  |
| No | 1272 (99.4) | 1269 (99.1) |  |  |
| Self-reported hypertension ^b^ |  |  | 7.761 | .005 ^d^ |
| Yes | 155 (12.1) | 205 (16.0) |  |  |
| No | 1125 (87.9) | 1075 (84.0) |  |  |
| Self-reported CVD ^b^ |  |  | 1.456 | .228 ^d^ |
| Yes | 37 (2.9) | 49 (3.8) |  |  |
| No | 1243 (97.1) | 1231 (96.2) |  |  |
| Self-reported cancer ^b^ |  |  | 0.072 | .789 ^d^ |
| Yes | 6 (0.5) | 8 (0.6) |  |  |
| No | 1274 (99.5) | 1272 (99.4) |  |  |
| Denture ^b^ |  |  | <0.001 | 1.000 ^d^ |
| Yes | 343 (26.8) | 342 (26.7) |  |  |
| No | 937 (73.2) | 938 (73.3) |  |  |

Abbreviations: CVD, coronary heart disease.

^a^Median (25th percentile, 75th percentile).

^b^n (%)

^c^Kruskal-Wallis H test

^d^Chi-square test
