# Supplementary material for: Edentulousness and the Likelihood of Becoming a Centenarian: Longitudinal Observational Study
Source: JMIR Aging. 2025 Mar 21;8:e68444. doi: 10.2196/68444 (PMC11951808; doi:10.2196/68444)
Supplement: Multimedia Appendix 2 [file aging-v8-e68444-s002.docx]

Multimedia Appendix2. Association between the number of natural teeth and becoming a centenarian.

|  | Model 1 |  | Model 2 |  | Model 3 |  |
| --- | --- | --- | --- | --- | --- | --- |
|  | OR (95%CI) | *P* Value | OR (95%CI) | *P* Value | OR (95%CI) | *P* Value |
| Teeth | 0.946 (0.932, 0.961) | <.001 | 1.019 (1.000, 1.038) | .051 | 1.022 (1.002, 1.042) | .028 |

Note: *P*<0.05 indicates statistical significance.

Abbreviations: OR: Odd ratio; CI: confidence interval; Ref: reference.

Note: Model 1-Unadjusted. Model 2-Model 1 additionally adjusted for the age based on the 1998 survey, sex, race, marital status, smoking status, alcohol use and exercise habit. Model 3-Model 2 plus additional adjustment for denture use and disease histories (including diabetes, hypertension, CVD, and cancer).
